# Assessment of Probable Opioid Use Disorder Using Electronic Health Record Documentation

**DOI:** 10.1001/jamanetworkopen.2020.15909

**Published:** 2020-09-04

**Authors:** Sarah A. Palumbo, Kayleigh M. Adamson, Sarathbabu Krishnamurthy, Shivani Manoharan, Donielle Beiler, Anthony Seiwell, Colt Young, Raghu Metpally, Richard C. Crist, Glenn A. Doyle, Thomas N. Ferraro, Mingyao Li, Wade H. Berrettini, Janet D. Robishaw, Vanessa Troiani

**Affiliations:** 1 Department of Biomedical Science, Schmidt College of Medicine of Florida Atlantic University, Boca Raton; 2 Geisinger Clinic, Geisinger, Danville, Pennsylvania; 3 Department of Molecular and Functional Genomics, Geisinger, Danville, Pennsylvania; 4 Center for Neurobiology and Behavior, Department of Psychiatry, University of Pennsylvania Perelman School of Medicine, Philadelphia; 5 Department of Biomedical Sciences, Cooper Medical School of Rowan University, Camden, New Jersey; 6 Department of Biostatistics, Epidemiology and Informatics, University of Pennsylvania Perelman School of Medicine, Philadelphia; 7 Department of Imaging Science and Innovation, Geisinger, Danville, Pennsylvania; 8 Neuroscience Institute, Geisinger, Danville, Pennsylvania; 9 Department of Basic Sciences, Geisinger Commonwealth School of Medicine, Scranton, Pennsylvania

## Abstract

**Importance:**

Electronic health records are a potentially valuable source of information for identifying patients with opioid use disorder (OUD).

**Objective:**

To evaluate whether proxy measures from electronic health record data can be used reliably to identify patients with probable OUD based on *Diagnostic and Statistical Manual of Mental Disorders* (Fifth Edition) (*DSM-5)* criteria.

**Design, Setting, and Participants:**

This retrospective cross-sectional study analyzed individuals within the Geisinger health system who were prescribed opioids between December 31, 2000, and May 31, 2017, using a mixed-methods approach. The cohort was identified from 16 253 patients enrolled in a contract-based, Geisinger-specific medication monitoring program (GMMP) for opioid use, including patients who maintained or violated contract terms, as well as a demographically matched control group of 16 253 patients who were prescribed opioids but not enrolled in the GMMP. Substance use diagnoses and psychiatric comorbidities were assessed using automated electronic health record summaries. A manual medical record review procedure using *DSM-5* criteria for OUD was completed for a subset of patients. The analysis was conducted beginning from June 5, 2017, until May 29, 2020.

**Main Outcomes and Measures:**

The primary outcome was the prevalence of OUD as defined by proxy measures for *DSM-5* criteria for OUD as well as the prevalence of comorbidities among patients prescribed opioids within an integrated health system.

**Results:**

Among the 16 253 patients enrolled in the GMMP (9309 women [57%]; mean [SD] age, 52 [14] years), OUD diagnoses as defined by diagnostic codes were present at a much lower rate than expected (291 [2%]), indicating the necessity for alternative diagnostic strategies. The *DSM-5* criteria for OUD can be assessed using manual medical record review; a manual review of 200 patients in the GMMP and 200 control patients identifed a larger percentage of patients with probable moderate to severe OUD (GMMP, 145 of 200 [73%]; and control, 27 of 200 [14%]) compared with the prevalence of OUD assessed using diagnostic codes.

**Conclusions and Relevance:**

These results suggest that patients with OUD may be identified using information available in the electronic health record, even when diagnostic codes do not reflect this diagnosis. Furthermore, the study demonstrates the utility of coding for *DSM-5* criteria from medical records to generate a quantitative *DSM-5* score that is associated with OUD severity.

## Key Points

**Question:**

Are medication monitoring programs within a hospital associated with more accurate identification of patients with opioid use disorder through the use of proxy *Diagnostic and Statistical Manual of Mental Disorders* (Fifth Edition) (*DSM-5)* criteria for opioid use disorder extracted from electronic health records?

**Findings:**

This cross-sectional study demonstrated that *DSM-5* criteria for opioid use disorder can be extracted through review of electronic health records and that patients who are part of a drug monitoring program had a higher mean prevalence of opiod use disorder and a higher mean number of psychiatric comorbidities associated with opioid use disorder.

**Meaning:**

Proxy measures that rely on multiple sources of data, including prescription drug history and notes in the electronic health record, may help identify patients with opioid use disorder who have not received a diagnosis.

## Teaser

This cross-sectional study evaluated whether proxy measures from electronic health record data can be used reliably to identify patients with probable opioid use disorder based on *Diagnostic and Statistical Manual of Mental Disorders* (Fifth Edition) criteria.

## Introduction

Opioid use disorder (OUD) is an epidemic that has been escalating in the United States for the past 2 decades. Although the rate of prescribing opioid analgesics has been decreasing since 2012,^1^ the number of synthetic opioid–related deaths has been exponentially increasing,^2^ and this trend is anticipated to continue. Most patients with OUD use heroin and/or fentanyl,^3,4,5,6^ but 50% to 90% of patients with OUD were exposed to a prescription opioid first.^7,8^ The prevalence estimate of OUD in the US in 2018 is 2 million individuals,^9^ similar to the previous year’s prevalence estimates.^10^ However, OUD is likely underdiagnosed within the health system setting. This underdiagnosis may be due, in part, to the reticence of practitioners who lack the specialized training in addiction medicine required to diagnose and treat OUD despite the fact that the most common source of opioid prescriptions is from primary care settings.^11^

Typically, OUD is diagnosed during a patient-physician consultation during which the addiction-trained practitioner uses dialogue with the patient or questionnaires to evaluate whether the patient exhibits symptoms of OUD based on *Diagnostic and Statistical Manual of Mental Disorders* (Fifth Edition) (*DSM-5*) OUD criteria. These criteria are based on the assessment of whether opioid use causes significant impairment in physical and social functioning, as well as aspects of craving and unsuccessful efforts to reduce or control use. The presentation of 2 or more of the 11 *DSM-5* criteria for OUD within a 12-month period warrants an OUD diagnosis. More important, the practitioner typically relies on the self-report of the patient but may consult a significant other or relative of the patient.

Electronic health record (EHR) data provide a wealth of information, including patients’ previous health care encounters, demographic characteristics, and prescription history. Within an integrated health care system, such as Geisinger, where patients seek primary and specialty care in the same network, these variables could be particularly important to consider for frequently underdiagnosed conditions, such as OUD.

The goal of this research is to use the comprehensive EHR data of patients who are prescribed opioids to develop proxy measures for the *DSM-5* criteria for OUD. To accomplish this, we used EHR data and a contract-based medication monitoring program that exists within Geisinger, a large, integrated health system. We hypothesized that patients who violated the terms of this contract would have more clinical characteristics of OUD compared with those who maintained their contract.

## Methods

### Data Sources and Patient Cohort

**Figure 1.**
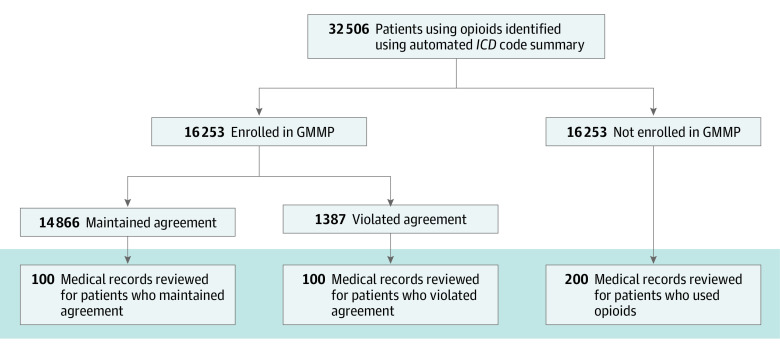
Flow Diagram Patients taking opioids in the context of cancer treatment and/or for hospice care were excluded prior to group identification. Shading indicates the subset of patients whose medical records were randomly selected for manual review. GMMP indicates Geisinger medication monitoring program; and *ICD*, *International Classification of Diseases, Ninth Revision* and/or *International Statistical Classification of Diseases and Related Health Problems, Tenth Revision*.

This retrospective, observational, cross-sectional study was implemented in 2 primary groups. We identified a large cohort of patients at Geisinger, an integrated health system in central Pennsylvania that has used the EPIC EHR system since 1996, who were treated with opioids for nonprogressive musculoskeletal pain. A subset of 400 patients was randomly selected from the cohort for a manual medical record review (Figure 1). The medical record review portion of this project was approved as human participant research by the Geisinger Institutional Review Board, and a waiver of Health Insurance Portability and Accountability Act authorization and research consent was granted owing to the retrospective nature of the study, absence of direct risk posed to the participants, and limited nature of the data set (small subsample included in the review). A second part of this study was designed to measure the prevalence of psychiatric and substance use phenotypes in both the medical record review and the larger cohort using automated EHR data extraction methods (ie, *International Classification of Diseases, Ninth Revision* [*ICD-9*] and *International Statistical Classification of Diseases and Related Health Problems, Tenth Revision* [*ICD-10*] codes). The automated data extraction portion of this project was deemed exempt by the Geisinger Institutional Review Board because all variables were extracted and deidentified using an approved data broker. This study followed the Strengthening the Reporting of Observational Studies in Epidemiology (STROBE) reporting guideline.

Geisinger instituted their own medication monitoring program (GMMP), aimed at standardizing opioid prescribing practices and enhancing patient adherence with medication instructions. The contract requires patients to submit to random urine drug screening tests and to take only the opioid and opioid dose prescribed by the designated Geisinger physician. Although use of the contract is encouraged by all physicians prescribing controlled substances for chronic pain, implementation of the contract is ultimately at the discretion of an individual clinician, and many patients are prescribed opioids without being enrolled in the GMMP. Documentation of program enrollment is recorded in EPIC with a Geisinger-specific code, along with a PDF file of the patient’s signed contract. Patients who violate the terms of the contract can be reported by the clinician and are designated in the EHR with another Geisinger-specific EPIC code. Full details of the contract are provided in the eAppendix in the Supplement. Because the contract is implemented based on physician discretion, there are a substantial number of patients prescribed opioids but not enrolled in the GMMP.

We analyzed 16 253 individuals aged 18 to 75 years enrolled in the GMMP for an opioid prescription between December 31, 2000, and May 31, 2017, using a mixed-methods approach. A Geisinger data broker was used to identify patients within the GMMP program (based on EPIC coding). The data broker also selected the matched (age, sex, and smoking history) population from a pool of all Geisinger patients who were not part of the GMMP and had at least 2 opioid prescriptions in their medication history. Patients with terminal illness and those with metastatic cancer were excluded from both groups prior to matching. We completed a medical record review on a subset of these patients, including 200 patients from the GMMP group (100 who maintained an active contract [GMMP-M] and 100 who had violated the contract [GMMP-V]) and 200 patients from the control group. In addition to the medical record review, we also assessed the clinical characteristics of both patient cohorts (N = 32 506) using *ICD-9* and *ICD-10* codes for psychiatric disorders and substance use.

### Outcomes

Our primary outcome was a quantification of OUD-related symptoms consistent with *DSM-5* criteria. Secondarily, we aimed to characterize psychiatric and addiction comorbidities within this population of patients using opioids.

### Demographic Factors and Disease Codes

The *ICD-9* and *ICD-10* codes associated with psychiatric disorders (including substance use) were drawn from patient problem lists and clinical encounters. All *ICD-9* and *ICD-10* codes and their relevant categories used in this research study are in eTable 1 in the Supplement. The *ICD-9* and *ICD-10* psychiatric codes were categorized into 1 of 6 diagnostic categories: depression, anxiety, tobacco use disorder, alcohol use disorder, OUD, and other substance use disorder. Patients were required to have at least 3 clinical encounters with notation of an *ICD-9* or *ICD-10* code to be characterized as having that disorder, consistent with previous work using *ICD* codes for phenotyping in other psychiatric disorders.^12^ All opioid prescription records were also drawn from the EHR, and the morphine milligram equivalent (MME) dose was calculated using the global rPH calculator.^13^ See the eAppendix in the Supplement for included medications.

### Medical Record Review Variable Selection and Recording

We characterized the clinical profile of a subset of patients receiving opioids using a manual medical record review procedure. Detailed information was extracted from the medical record for each patient, including variables associated with the GMMP agreement, such as termination cause, as well as other quantitative phenotypes, such as opioid MME, toxicology screening test pass or fail data, and number of emergency department visits (see eAppendix in the Supplement for search terms). We also adapted criteria from the *DSM-5*^14^ to diagnose OUD using EHR search and review (Table 1). We excluded criteria associated with opioid tolerance and withdrawal because these criteria are based on physical dependence, which will affect any patient with long-term opioid use. It was then determined by expert clinician review (W.H.B.) whether each medical record review variable was relevant for each of the 9 *DSM-5* criteria evaluated here (Table 1). After review of each patient’s medical record, the presence or absence of a given EHR search variable or behavior was recorded. The medical record reviewer was not blinded to the patient’s status in the program because it was not possible given the nature of the comprehensive manual medical record review. A score was generated in which the patient received 1 point for each of the 9 *DSM-5* criteria satisfied. These scores were then stratified into the 4 standardized categories from the *DSM-5*: no OUD (scores <2), mild OUD (scores 2-3), moderate OUD (scores 4-5), and severe OUD (scores ≥6).

**Table 1.** Electronic Health Record Search Categories Defined for Identifying 9 *DSM-5* Criteria for OUD

Category	*DSM-5* criteria^a^								
	1	2	3	4	5	6	7	8	9
Vocational interference owing to drug use or pain	No	No	No	No	Yes	No	Yes	No	Yes
Disabled	No	No	No	No	Yes	Yes	Yes	No	No
Weaning described as unsuccessful or difficult	No	Yes	No	No	No	No	No	No	No
Positive toxicology screening test result for opioids other than prescribed	Yes	Yes	Yes	Yes	No	No	No	No	Yes
Lost pills	Yes	No	Yes	No	No	No	No	No	No
Multiple opioid prescribers	Yes	No	Yes	No	No	No	No	No	No
Multiple pharmacies	Yes	No	Yes	No	No	No	No	No	No
Early prescription refills	Yes	Yes	No	No	No	No	No	No	No
Opioid overdose	No	No	No	No	No	No	No	Yes	Yes
Substance abuse	Yes	No	No	No	No	No	No	No	Yes
Hazardous situation as result of opioid	No	No	No	No	No	No	No	Yes	No
Interpersonal or legal issues as result of opioid	No	No	No	No	Yes	Yes	Yes	No	No
Medical issues as result of opioid	No	No	No	No	Yes	No	No	No	Yes
Craving	No	Yes	No	Yes	No	No	No	No	No
Clinician mentioned drug-seeking behavior	Yes	Yes	Yes	Yes	No	No	No	No	Yes

Abbreviations: *DSM-5*, *Diagnostic and Statistical Manual of Mental Disorders* (Fifth Edition); OUD, opioid use disorder.

^a^ Criteria for OUD: (1) more or longer use of opioids than intended, (2) unsuccessful efforts to cut down use, (3) time taken to obtain opioids or recover from opioid use, (4) craving, (5) effect on work or school, (6) effect on interpersonal relationships, (7) reduced activities because of use, (8) continued use when physically hazardous, and (9) use despite physical or psychological problems.

### Statistical Analysis

Statistical analysis was conducted from June 5, 2017, to May 29, 2020. Summary scores were compared between the GMMP and control groups, and within the GMMP group between the GMMP-M and GMMP-V groups. For medical record review comparisons, differences in the frequency of individuals with each OUD severity were assessed using χ^2^ tests. For both the medical record review cohort and the larger group (N = 32 506), *ICD-9* and *ICD-10* codes were extracted from the EHR and aggregated across associated psychiatric and addiction categories to limit multiple hypothesis testing and improve power. The percentages of individuals expressing each phenotype variable were compared between the GMMP and control groups with proportion tests using the “stats” package in R Studio.^15^ All *P* values were from 2-sided tests, and results were deemed statistically significant at *P* < .05.

## Results

### OUD Diagnoses

A total of 16 253 patients (9309 women [57%]; mean [SD] age, 52 [14] years) were enrolled in the GMMP. Among these patients, OUD diagnoses were present at a much lower rate than expected (291 [2%]), indicating the necessity for alternative diagnostic strategies.

### Record Review

**Figure 2.**
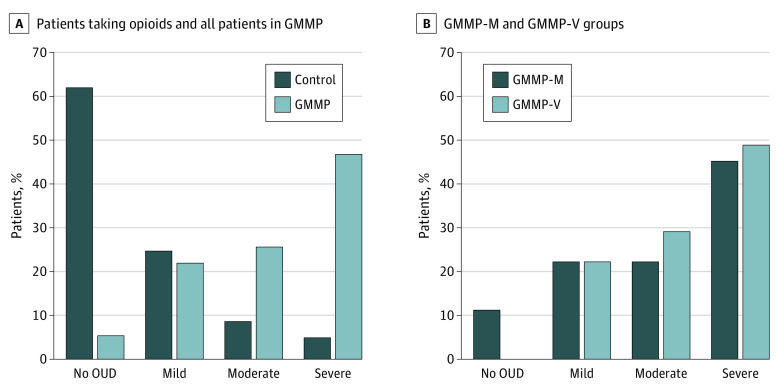
Percentage of Patients With Each *Diagnostic and Statistical Manual of Mental Disorders* (Fifth Edition) (*DSM*) Severity Score From Medical Record Review A, Percentage of patients with each *DSM* severity score from medical record review for opioid-exposed controls (n = 200) and all patients in the Geisinger medication monitoring program (GMMP) (n = 200). B, Percentage of patients with each *DSM* severity score from medical record review for those enrolled in the GMMP who maintained the contract (GMMP-M; n = 100) and those enrolled in the GMMP who violated the contract (GMMP-V; n = 100). OUD indicates opioid use disorder.

The medical records of 200 patients in the GMMP group (100 in GMMP-M and 100 in GMMP-V) were reviewed for *DSM-5* criteria for OUD. The results of the *DSM-5* criteria medical record review indicated that 67 of 100 GMMP-M patients whose medical record was reviewed (67%) and 78 of 100 GMMP-V patients whose medical record was reviewed (78%) were classified as having moderate to severe OUD (χ^2^_1_ = 3.03; n = 200; *P* = .08). These scores are presented in Figure 2A. Violations of the contract leading to GMMP-V status were predominantly based on failed urine toxicology screening tests that were positive for unprescribed medication (64 of 100 [64%]) or negative for prescribed medications (48 of 100 [48%]), with many patients both positive for unprescribed medications and negative for prescribed medications (35 of 100 [35%]). We also identified many patients in the GMMP-M group who exhibited behaviors that violated contract terms, including urine toxicology screening test results that were positive for unprescribed medications (20 of 100 [20%]), negative for prescribed medications (17 of 100 [17%]), or both (10 of 100 [10%]). More important, 66 of 100 patients (66%) in the GMMP-M group had at least 1 documentation that indicated a contract violation (for numbers of each patient group with each violation and toxicology screening test data, see eTables 3, 4, and 5 in the Supplement). Given the large number of patients who maintained their contract despite behaviors consistent with contract violations, we then collapsed the GMMP-V and GMMP-M populations into 1 GMMP medical record review population (n = 200) and compared that GMMP group with a separate control group of patients who were prescribed opioids but who were not in the GMMP (n = 200). In the control group, 27 patients (14%) were classified as having moderate to severe OUD, compared with 145 patients (73%) in the GMMP population (χ^2^_1_ = 223.9; n = 400; *P* < .001; Figure 2B).

Patients in the combined medical record review GMMP group also had higher rates of depression (82 of 200 [41%] vs 33 of 200 [16%]; *P* < .001) and anxiety (92 of 200 [46%] vs 39 of 200 [20%]; *P* < .001), as well as greater nicotine use (85 of 200 [42%] vs 53 of 200 [26%]; *P* < .001), opioid use (6 of 200 [3%] vs 0; *P* = .04), and other substance use (9 of 200 [4%] vs 1 of 200 [1%]; *P* = .03) relative to the control population (Table 2). In addition, patients in the combined GMMP group sought out treatment in the emergency department more frequently than patients in the control group (13 vs 5 visits; *P* = .007).

**Table 2.** Demographic and Health Record Summary Data for Health Record Review Subsample

Sample demographic data	GMMP group (n = 200)	Control group (n = 200)	*P* value	Test statistic	Effect size (95% CI)
Sex, No. (%)					
Male	84 (42)	79 (40)	.61	−0.51	0.03 (0.072-0.122)
Female	116 (58)	121 (60)			
Age, mean (SD), y	48.1 (10.5)	48.2 (10.7)	.89	−0.13	0.14 (1.94-2.22)
BMI, mean (SD)	31 (8)	31 (8)	.59	−0.54	0.44 (1.16-2.04)
Health record data, mean (SD)					
EHR length, d	4076 (1902)	3829 (1843)	.31	1.01	211 (198-620)
No. of ED visits	13 (33)	5 (6)	.007	2.75	8.16 (2.29-14.01)
Daily MME	52 (35)	36 (19)	.10	1.91	16.0 (3.97-35.8)
Psychiatric codes, No. (%)					
Depression	82 (41)	33 (16)	<.001	28.1	0.245 (0.155-0.335)
Anxiety	92 (46)	39 (20)	<.001	30.7	0.265 (0.172-0.358)
Depression and anxiety	52 (26)	15 (8)	<.001	23.2	0.185 (0.109-0.261)
Addiction codes					
Alcohol	7 (4)	3 (2)	.34	0.9	0.015 (0.016-0.056)
Nicotine	85 (42)	53 (26)	<.001	10.6	0.16 (0.063-0.257)
Opioids	6 (3)	0	.04	NA	0.03 (0.001-0.059)
Other substance abuse	9 (4)	1 (1)	.03	5.0	0.04 (0.005-0.075)
Alcohol, nicotine, opioids, or other	0	0	NA	NA	NA

Abbreviations: BMI, body mass index (calculated as weight in kilograms divided by height in meters squared); ED, emergency department; EHR, electronic health record; GMMP, Geisinger-specific medication monitoring program; MME, morphine milligram equivalent; NA, not applicable.

### Large Cohort Phenotype Comparisons of *ICD-9* and *ICD-10* Codes

Since the medical record review results indicated that a substantial percentage of patients in the GMMP group showed signs of OUD, we compared demographic differences and psychiatric and addiction diagnosis differences between the entire GMMP population (n = 16 253) and controls (n = 16 253) (Table 3). Within the combined GMMP group, we found higher rates of depression (5446 [34%] vs 1473 [9%]; *P* < .001) and anxiety (6552 [40%] vs 1605 [10%]; *P* < .001). We also found higher rates of tobacco use (4760 [29%] vs 1523 [9%]; *P* < .001), alcohol use (489 [3%] vs 137 [1%]; *P* < .001), and other substance use (570 [4%] vs 106 [1%]; *P* < .001) compared with the opioid-treated control group. Similar differences in psychiatric and substance use codes were observed in the subset of patients assessed using the medical record review procedure (Table 2) and when comparing the larger group of patients in the GMMP-V group with the patients in the GMMP-M group (eTables 2 and 3 in the Supplement).

**Table 3.** Demographic and Health Record Summary Data for Entire Opioid-Using, Geisinger-Specific Medication Monitoring Program and Control Population

Sample demographic data	GMMP group (n = 16 253)	Control group (n = 16 253)	*P* value	Test statistic	Effect size (95% CI)
Sex, No. (%)					
Male	6944 (43)	6949 (43)	.93	−0.1	0.00 (0.010-0.011)
Female	9309 (57)	9304 (57)			
Age, mean (SD), y	52 (14)	50 (14)	<.001	12.0	1.88 (1.57-2.19)
BMI, mean (SD)	32 (8)	32 (8)	.01	2.6	0.24 (0.06-0.42)
Health record data, mean (SD)					
EHR length, d	4211 (2073)	2650 (2352)	<.001	63.2	1560 (1512-1608)
No. of ED visits	8.6 (16.0)	3.6 (5.0)	<.001	29.4	4.99 (4.65-5.31)
Daily MME	52 (78)	44 (72)	<.001	9.2	7.41 (5.82-8.99)
Psychiatric codes, No. (%)					
Depression	5446 (34)	1473 (9)	<.001	2897	0.245 (0.236-0.253)
Anxiety	6552 (40)	1605 (10)	<.001	4004	0.304 (0.296-0.313)
Depression and anxiety	3434 (21)	663 (4)	<.001	2143	0.171 (0.163-0.178)
Addiction codes, No. (%)					
Alcohol	489 (3)	137 (1)	<.001	201	0.022 (0.019-0.025)
Nicotine	4760 (29)	1523 (9)	<.001	2066	0.199 (0.191-0.208)
Opioids	291 (2)	48 (0.3)	<.001	175	0.015 (0.013-0.017)
Other substance abuse	570 (4)	106 (1)	<.001	324	0.029 (0.025-0.0317)
Alcohol, nicotine, opioids, or other	14 (0.1)	5 (0.03)	.03	5	0.000615 (0.00004-0.0011)

Abbreviations: BMI, body mass index (calculated as weight in kilograms divided by height in meters squared); ED, emergency department; EHR, electronic health record; GMMP, Geisinger-specific medication monitoring program; MME, morphine milligram equivalent.

## Discussion

We found that patients enrolled in a contract-based, health system–specific drug monitoring program showed higher rates of OUD based on a medical record review procedure that adapted *DSM-5* interview criteria. We also observed that when patients are appropriately documented as having violated the terms of the contract with an EPIC code, this code can be a useful proxy for OUD diagnosis. This finding is consistent with previous work that demonstrated the utility of a prescription monitoring program.^16^

We used a manual medical record review procedure but searched for consistent and predefined search terms. This method is different from natural language processing algorithms, as a human reviewer can interpret whether certain search terms are appropriate in a given context. For example, for “substance abuse mentioned,” we included search terms such as “abuse” and “high.” A human reviewer can evaluate if those search terms appear in a context relevant to drug abuse. These search terms could serve as the basis of future natural language processing algorithms and would improve the scalability of this method. Future work may also benefit from combining search terms and *ICD* codes, as Carell et al^17^ reported that the combined use of *ICD* codes and natural language processing data were more effective in identifying OUD than either method alone. Other work using natural language processing has shown that more than one-third of patients with inappropriate opioid use in the setting of chronic pain did not have an *ICD* code associated with their opioid misuse.^18^ This finding is consistent with our finding that very few patients had *ICD-9* or *ICD-10* codes for OUD.

We also demonstrate that psychiatric and other substance use codes are increased in patients in the drug monitoring program. This finding is consistent with epidemiologic data of OUD^14^ and other work in chronic pain populations.^19^ These results suggest the potential for assessing psychiatric and other substance use codes as an associated factor to evaluate patient risk for OUD in the chronic pain setting. Others have also confirmed the utility of assessing EHRs of patient populations to identify risk factors, such as comorbidities and illicit drug use, associated with opioid misuse and overdose.^16,20^

Studies have described clinical characteristics of patients with pain who are at risk for developing prescription opioid addiction in the context of chronic opioid treatment of persistent pain.^21,22^ Factors associated with increased risk of OUD in cross-sectional studies include younger age, male sex, European ancestry, comorbid psychiatric disorders, higher MME, lower socioeconomic and educational achievement, exposure to violence or sexual assault, inability to work owing to disability, and a personal or family history of substance use disorders.^21,22,23,24,25,26^ A previous study of OUD among 705 Geisinger patients taking prescription opioids for at least 5 months in a 12-month period for noncancer pain revealed that the characteristics most frequently associated with severe OUD are age younger than 65 years, current pain impairment, sleep disturbance, suicidal thoughts, anxiety disorders, and history of substance abuse with treatment.^21^ In that study, approximately 13% of these 705 patients satisfied psychiatric-based criteria for moderate to severe OUD.

Data from the present study are also relevant to the overall risk for developing OUD when being treated for chronic pain. Estimates for OUD in the context of treatment for chronic pain vary widely, with estimates ranging from less than 10% to more than 25%.^21,27,28,29^ Our estimate of OUD in controls treated with opioids (14%) is consistent with the lower end of this estimate but is much higher for those in the GMMP (73%). This difference may be, in part, because physicians are more likely to refer someone to the GMMP based on an increased suspicion that the prescribed opioid is being misused or owing to closer monitoring of patients once they are enrolled in the program. There may also be a bias toward identifying OUD once the patient is in the program given the increased monitoring of program participants.

### Limitations

This study has some limitations. It relies on EHR data, which are dependent on physicians’ entries; thus, the information may not be standardized or available for all candidates. Some relevant items might also not be included in the EHR. For example, family history of substance use disorders is poorly captured despite being a known risk factor for developing OUD.^30,31,32,33^ We also did not assess differences in nonpsychiatric comorbidities, such as pain. Future work would benefit from assessing whether certain types of pain are associated with increased risk for OUD. In addition, this study was conducted in a single health care system and thus may have certain population characteristics that are unique and may be associated with the results of the study. Future work should explore the validity of applying this medical record review estimation of OUD severity in other health systems.

Prescription and claims data are also thought to underestimate opioid use.^34^ These data might result in underestimates of MME for patients who are seeking additional prescriptions outside of Geisinger. Others have been successful in incorporating prescription data from national databases to assess more comprehensive OUD phenotypes surrounding prolonged use of opioids in the context of chronic pain and after surgery.^18,35,36,37,38,39,40^ Future work should incorporate additional prescription databases, when available. At the time of this study, the Pennsylvania State Drug Monitoring Program was not available for research use.

Some of the *DSM-5* criteria used in this study may not map directly from a more traditional interview format to those adapted to available EHR data. For example, we used disability status as a proxy for *DSM-5* criteria including “work/school impact,” “interpersonal impact,” and “reduced activities because of use.” A person can become disabled for many reasons, including chronic pain and/or other injury not associated with opioid misuse. We chose to generate severity scores that would closely mirror the traditional interview format, but future iterations of this work may find it more useful to characterize severity based on the presence of an individual search term or a subset of search terms.

Many of the individuals in the GMMP program had diagnosis codes for other substance use disorders and other psychiatric disorders. Assessing whether a psychiatric illness came before or after opioid use is challenging with EHR data, as the date of the first recorded diagnosis does not necessarily co-occur with the onset of the disorder. Future work should explore the temporality of medication use and diagnoses and/or explore different trajectories associated with distinct patient subgroups.

Other work has used *ICD* codes and other EHR data to characterize various opioid use phenotypes. For example, health record data have been used to assess which patients go on to develop chronic use of opioids using opioid-related search terms, emergency department chief symptoms, prescription history, and other variables.^41,42,43,44^ Future work is needed to develop the most optimized algorithm for use across multiple health records.

## Conclusions

We show in the present study that EHRs can be used to derive *DSM-5* severity scores for OUD. Our methods are unique in deriving a severity score that aims to mirror severity scores from more traditional interview-based diagnostic procedures, but results are also consistent with previous work examining OUD in EHR data. Thus, this study contributes to the growing body of knowledge that emphasizes the utility of EHRs to evaluate a patient’s status or potential for opioid or other substance misuse. Opioids continue to be used for the treatment of pain. Precision medicine within integrated health systems such as Geisinger could be a major associated factor in developing more efficient pain treatments with less risk for addiction, and studies of this potential could be helped by establishing more effective proxy measures for OUD using EHR data.

## Notes

Supplement. **eAppendix.** Supplemental Methods **eTable 1.** *ICD 9/10* Codes Used for Each Phenotype **eTable 2.** Demographic and Health Record Summary Data for GMMP-M and GMMP-V Subgroups **eTable 3.** EHR Search Categories and Number of Patients With Each Category **eTable 4.** Specific Toxicology Screen Violations by Subtype **eTable 5.** Frequency of Positive Search Criteria
