# Supplementary material for: Assessment of Probable Opioid Use Disorder Using Electronic Health Record Documentation
Source: JAMA Netw Open. 2020 Sep 4;3(9):e2015909. doi: 10.1001/jamanetworkopen.2020.15909 (PMC7489858; doi:10.1001/jamanetworkopen.2020.15909)
Supplement: Supplement. — eAppendix. Supplemental Methods eTable 1. ICD 9/10 Codes Used for Each Phenotype eTable 2. Demographic and Health Record Summary Data for GMMP-M and GMMP-V Subgroups eTable 3. EHR Search Categories and Number of Patients With Each Category eTable 4. Specific Toxicology Screen Violations by Subtype eTable 5. Frequency of Positive Search Criteria [file jamanetwopen-e2015909-s001.pdf]

Supplementary Online Content

Palumbo SA, Adamson KM, Krishnamurthy S, et al. Assessment of probable opioid use disorder using electronic health record documentation. *JAMA Netw Open.* 2020;3(9):e2015909. doi:10.1001/jamanetworkopen.2020.15909

eAppendix. Supplemental Methods
eTable 1. *ICD 9/10* Codes Used for Each Phenotype
eTable 2. Demographic and Health Record Summary Data for GMMP-M and GMMP-V Subgroups
eTable 3. EHR Search Categories and Number of Patients With Each Category
eTable 4. Specific Toxicology Screen Violations by Subtype
eTable 5. Frequency of Positive Search Criteria

This supplementary material has been provided by the authors to give readers additional information about their work.

© 2020 Palumbo SA et al. *JAMA Network Open.*

eAppendix. Supplemental Methods

eMethods 1: Agreement terms of the Patient Drug Monitoring Program contracts.
Patients that sign this contract agree to the following terms:
(1) To attend regularly scheduled appointments.
(2) To take medication only as prescribed.
(3) To agree to one provider and fills at one specified pharmacy.
(4) To pick up own prescription at pharmacy or by authorized persons (named in agreement).
(5) To not to obtain other controlled substances, including opioid pain medicines, controlled stimulants or anti-anxiety medicines from any other provider.
(6) To not borrow or accept medication from friends, use street or illegal drugs, and/or sell/give medication to another person.
(7) To not make any attempt to forge or alter any prescriptions.
(8) To safeguard medications to prevent lost or theft.
(9) To submit to random drug screens and pill counts.

Patients who violate the terms of this agreement are denoted with a separate EPIC code.

Examples of violations of the above are as follows:
(1) Cancellation or no show for two or more appointments (considered a cancellation if less than one working days' notice).
(2) Increasing or decreasing dose without discussing with provider.
(3) Seeking and/or filling prescription(s) from another provider or multiple pharmacies.
(4) Allowing someone other than self or authorized person to pick up prescription.
(5) Obtaining other controlled substances, including those legally prescribed by other physicians and/or illegal acquisition of prescription or street drugs.
(6) To forge or alter prescriptions.
(7) Repeated reports of lost or stolen medication.
(8) Refusal to submit to urine toxicology screen and/or pill counts.
(9) Positive urine toxicology screen for substances not prescribed.

eMethods 2: Opioid drugs included and equivalent dose determination:
All opioid prescription records were also drawn from the EHR, and morphine milligram equivalent (MME) dose was calculated using the global rPH calculator (http://www.globalrph.com/narcotic.cgi). Opioid drugs included were hydromorphone, methadone, oxymorphone, oxycodone, morphine sulfate, hydrocodone, tapentadol, tramadol, codeine, and fentanyl.

eMethods 3: Chart Review Variables:
(A) Demographic and health record information relevant to the GMMP program for each group, included:

- (A1) Length of Electronic Health Record (EHR),
  The duration of the patient's health record was quantified in number of days. This was determined by calculating the number of days between the patient's very first encounter with the health system and the most recent visit, as of May 31, 2018.
- (A2) Current opioid prescription with Morphine Milligram Equivalence (MME),

© 2020 Palumbo SA et al. *JAMA Network Open.*

Current opiate prescription and dosage, and other controlled substances were recorded. Morphine milligram equivalence (MME) was calculated based on current prescription. Current prescriptions are located under the medications tabs. To identify current opioid prescriptions, the medication list was filtered by pharmaceutical class to only show analgesic narcotics. This same process was conducted to identify current controlled substance. To do so, the medication list was filtered by class and then by class II, III, and IV. MME was calculated using standard conversion procedure.

- (A3) Number of Emergency Room (ER) visits over the course of the patient's record:
  Emergency rooms visits were searched and number summed from the beginning of the patient's health record until their most recent visit.

(B) Criteria related to an OUD DSM diagnosis:

- (B1) Vocational interference due to drug use or pain
  A positive vocational interference may indicate that the patient is unable to work due to pain, or that their prescription opioid use is impacting their work. Health records were searched for terms that indicated if a patient's occupation was impacted by their use of opioids or pain. Terms that were searched included: occupation, work, job, boss, manager, too much pain, can't work.
- (B2) Patient Disability Status
  Health records, including notes and encounters were searched for terms that indicated whether the patient was disabled. A positive disability status may indicate that the patient's pain was so great that it interfered with their occupation. If it was mentioned that a patient was planning to file for disability or was waiting to hear if they were approved, they were categorized as a no. Terms that were searched included: disability, too much pain, unemployment, can't work, paperwork.
  -Yes- disability indicated in the EHR
  -No- Explicitly states the patient is not disabled
  -No Record- No mention of ability status
- (B3) Whether weaning was described as unsuccessful or difficult
  If it was indicated that the patient had been weaned off an opioid, a further search was conducted to determine if the weaning was difficult or had complications.
  -Yes- the record indicates that weaning was difficult or unsuccessful for the individual
  -No- weaning was mentioned for the individual but was successful and/or not difficult and complicated
  -No record- weaning was mentioned though the success and difficulty of the action was not recorded
  - N/A- weaning was not mentioned for this individual thus cannot be difficult or unsuccessful
- (B4) Positive toxicology screen for opioids other than prescribed
  In order to verify that the patient was following the conditions of the GMMP agreement, health records were searched for urine toxicology screens to ensure that medications that the patient was prescribed were present in their urine and non-prescribed medications were not present. Number of toxicology screens varied between patients ranging from no toxicology screens to 50+. Toxicology screens were identified under the lab tests section. Because there are several tests, a filter was used to identify toxicology screens. Tests that were searched were: toxicology screens, urine toxicology screens, and opiates

© 2020 Palumbo SA et al. *JAMA Network Open.*

confirmatory. The only toxicology screens that were reviewed were those that were collected during the GMMP agreement period. After the toxicology screen result was identified, prescribed medications at the time of the collection were verified. To do so, the medication list was opened and filtered by pharmaceutical class then by analgesics-narcotics. The date of the toxicology screen was identified and then matched to the closest date of prescription. The same process was conducted for controlled substances. The date of the toxicology screen was also identified if a medication was not present in the system in order to review the medications that we prescribed at that time.

- (B5) Lost pills mentioned

  Several scenarios consistently occurred in the chart review where patients reported missing pills such as having them stolen, misplaced, lost, or ruined due to accidents such as dropping them in water. Terms that were searched included: lost, missing, stolen, misplace, robbed, police report, and gone.
  -Yes- the record mentions lost pills
  -No- there was no mention of lost pills
- (B6) Multiple Opioid Prescribers Mentioned

  To search if a patient was receiving more than one opioid prescription, the medication list was searched for more than one providers or prescription during the same time period. If a patient received an opioid prescription from the emergency room while actively being prescribed an opioid, that was identified as multiple prescribers. If it was not evident in the medication list that the patient was receiving multiple prescriptions, terms were searched in the notes to identify if a prescriber identified that the patient was receiving more than one prescription or going to the more than one pharmacy. Terms that were searched included: multiple, various, pharmacy, and prescribers.
  -Yes- Multiple opioid prescribers were mentioned in the record
  -No- Multiple opioid prescribers were not mentioned in the record
- (B7) Multiple Pharmacies Mentioned

  To search if a patient was receiving more than one opioid prescription, the medication list was searched for more than one provider or prescription during the same time period. If a patient received an opioid prescription from the emergency room while actively being prescribed an opioid, that was identified as multiple prescribers. If it was not evident in the medication list that the patient was receiving multiple prescriptions, terms were searched in the notes to identify if a prescriber identified that the patient was receiving more than one prescription or going to the more than one pharmacy. Terms that were searched included: multiple, various, pharmacy, and prescribers.
  -Yes- Multiple Pharmacies were mentioned
  -No- Multiple Pharmacies were not mentioned
- (B8) Early Prescription Refills Mentioned

  Was it ever mentioned in the patient's health record if the patient requested an early an medication refill? Terms that were searched included: refill, too soon, early, and ran out.
  -Yes- Early refills were mentioned
  -No- Early refills were not mentioned
- (B9) Opioid overdose mentioned

  Terms that were searched included: overdose, intoxicated, unconscious, and intravenous.
  -Yes- Opioid overdose was mentioned
  -No- Opioid overdose was not mentioned

© 2020 Palumbo SA et al. *JAMA Network Open.*

- (B10) Substance abuse mentioned

  The patient was classified as "yes" for substance if illicit drugs that were not separately screened for (i.e. alcohol, marijuana, tobacco), were in the health record. Terms that were searched included: abuse, substance abuse, heroin, street drugs, detox, selling, dealing, and high.

  -Yes- substance abuse was mentioned

  -No- substance abuse was not mentioned
- (B11) Hazardous situation as a result of opioid mentioned

  An instance was considered to be hazardous if there was a suicide attempts via overdose, selling prescription, or ingesting prescription in conjunction with illicit drugs such as cocaine.

  -Yes- such a situation was noted in the record

  -No- a hazardous situation due to the opioid was not mentioned
- (B12) Interpersonal or legal issues as a result of opioid mentioned

  An instance was considered to be an interpersonal or legal issue if the police were involved in any capacity, if lawyers were mentioned, if jail or prison occurred, or if children services were mentioned.

  -Yes- issues were mentioned

  -No- issues were not mentioned
- (B13) Medical issues as a result of opioid mentioned

  Medical issues that were considered included narcotic abuse or dependence, withdrawal symptoms, constipation secondary to opioid use, or diseases contracted through drug use such as hepatitis C.

  -Yes- medical issues due to the opioid were mentioned

  -No- medical issues due to the opioid were not mentioned
- (B14) Craving mentioned

  Was it ever mentioned in the health record if the patient exhibited craving? The health record was first searched for the SOAPP-R (Screener and Opioid Assessment for Patients with Pain-Revised) which provides a risk score for opioid abuse. A patient was considered to exhibit craving symptoms if it was mentioned in their record that they were experiencing withdrawal symptoms or if it was specifically mentioned that they were craving the drug.

  -Yes- craving was mentioned

  -No- craving was not mentioned
- (B15) Provider mentioned drug-seeking behavior

  Was it ever mentioned in the health record if the patient exhibited drug-seeking behavior? The health record was first searched for the SOAPP-R (Screener and Opioid Assessment for Patients with Pain-Revised) which provides a risk score for opioid abuse. A patient was considered to exhibit drug-seeking behavior if it was mentioned in the physician's notes as so. Terms that were searched from drug-seeking behavior included: seeking, narcotic seeking, and drug seeking.

  -Yes- drug-seeking behavior was mentioned

  -No- drug-seeking behavior was not mentioned

© 2020 Palumbo SA et al. *JAMA Network Open.*

eTable 1. *ICD 9/10* Codes Used for Each Phenotype

ICD 9	*ICD 10*	Diagnosis
**Anxiety**		
300	F41.9	Anxiety disorder, unspecified
300.01	F41.0	Panic disorder [episodic paroxysmal anxiety] without agoraphobia
300.02	F41.1	Generalized anxiety disorder
300.09	F41.3	Other mixed anxiety disorders
300.09	F41.8	Other specified anxiety disorders
**Depression**		
296.3	F33.40	Major depressive disorder, recurrent, in remission, unspecified
296.3	F33.9	Major depressive disorder, recurrent, unspecified
296.31	F33.0	Major depressive disorder, recurrent, mild
296.32	F33.1	Major depressive disorder, recurrent, moderate
296.33	F33.2	Major depressive disorder, recurrent severe without psychotic features
296.34 or 298.0	F33.3	Major depressive disorder, recurrent, severe with psychotic symptoms
296.35	F33.41	Major depressive disorder, recurrent, in partial remission
296.36	F33.42	Major depressive disorder, recurrent, in full remission
296.99	F33.8	Other recurrent depressive disorders
311	F32.9	Depressive disorder, not elsewhere classified
**Alcohol**		
291	F10.121	Alcohol abuse with intoxication delirium
291	F10.231	Alcohol dependence with withdrawal delirium
291.3	F10.251	Alcohol dependence with alcohol-induced psychotic disorder with hallucinations
291.4	F10.929	Alcohol use, unspecified with intoxication, unspecified
291.81	F10.230	Alcohol dependence with withdrawal, uncomplicated
291.81	F10.239	Alcohol dependence with withdrawal, unspecified
291.89	F10.159	Alcohol abuse with alcohol-induced psychotic disorder, unspecified
291.89	F10.288	Alcohol dependence with other alcohol-induced disorder
291.9	F10.19	Alcohol abuse with unspecified alcohol-induced disorder
291.9	F10.29	Alcohol dependence with unspecified alcohol-induced disorder
291.9	F10.99	Alcohol use, unspecified with unspecified alcohol-induced disorder
303	F10.229	Acute alcoholic intoxication in alcoholism, unspecified
303.01	F10.229	Acute alcoholic intoxication in alcoholism, continuous
303.9	F10.20	Other and unspecified alcohol dependence
303.9	F10.20	Other and unspecified alcohol dependence, unspecified
303.9	F10.20	Alcohol dependence, uncomplicated
303.91	F10.20	Other and unspecified alcohol dependence, continuous

© 2020 Palumbo SA et al. *JAMA Network Open.*

303.92	F10.20	Other and unspecified alcohol dependence, episodic
303.93	F10.21	Other and unspecified alcohol dependence, in remission
303.93	F10.21	Alcohol dependence, in remission
305	F10.10	Alcohol abuse, unspecified
305.01	F10.10	Alcohol abuse, continuous
305.02	F10.10	Alcohol abuse, episodic
305.03	F10.11	Alcohol abuse, in remission
303 or 303.01	F10.229	Alcohol dependence with intoxication, unspecified
303.00 or 303.01 or 303.02 or 303.03	F10.220	Alcohol dependence with intoxication, uncomplicated
305 or 305.01 or 305.02	F10.10	Alcohol abuse, uncomplicated
305.00 or 305.01 or 305.02	F10.120	Alcohol abuse with intoxication, uncomplicated
305.00 or 305.01 or 305.02	F10.129	Alcohol abuse with intoxication, unspecified
**Nicotine**		
292.89	F17.218	Nicotine dependence, cigarettes, with other nicotine-induced disorders
292.89	F17.228	Nicotine dependence, chewing tobacco, with other nicotine-induced disorders
292.9	F17.209	Nicotine dependence, unspecified, with unspecified nicotine-induced disorders
292.9	F17.219	Nicotine dependence, cigarettes, with unspecified nicotine-induced disorders
292.9	F17.229	Nicotine dependence, chewing tobacco, with unspecified nicotine-induced disorders
305.1	F17.200	Tobacco use disorder
305.1	F17.200	Nicotine dependence, unspecified, uncomplicated
305.1	F17.201	Nicotine dependence, unspecified, in remission
305.1	F17.210	Nicotine dependence, cigarettes, uncomplicated
305.1	F17.220	Nicotine dependence, chewing tobacco, uncomplicated
305.1	F17.290	Nicotine dependence, other tobacco product, uncomplicated
*	F17.21	Nicotine dependence, cigarettes
**Opioid**		
304.7	*	Combinations of opioid type drug with any other drug dependence, unspecified
304.71	*	Combinations of opioid type drug with any other drug dependence, continuous
304.73	*	Combinations of opioid type drug with any other drug dependence, in remission
304.8	*	Combinations of drug dependence excluding opioid type drug, unspecified
304.81	*	Combinations of drug dependence excluding opioid type drug, continuous
304.82	*	Combinations of drug dependence excluding opioid type drug, episodic

© 2020 Palumbo SA et al. *JAMA Network Open.*

304.83	*	Combinations of drug dependence excluding opioid type drug, in remission
305.5	F11.10	Opioid abuse, unspecified
305.51	F11.10	Opioid abuse, continuous
305.52	F11.10	Opioid abuse, episodic
305.53	F11.11	Opioid abuse, in remission
**Other Substance Abuse**		
292	F15.93	Other stimulant use, unspecified with withdrawal
292	F19.930	Other psychoactive substance use, unspecified with withdrawal, uncomplicated
292	F19.939	Other psychoactive substance use, unspecified with withdrawal, unspecified
292.11	F19.950	Other psychoactive substance use, unspecified with psychoactive substance-induced psychotic disorder with delusions
292.12	F19.951	Other psychoactive substance use, unspecified with psychoactive substance-induced psychotic disorder with hallucinations
292.2	F12.120	Cannabis abuse with intoxication, uncomplicated
292.2	F15.920	Other stimulant use, unspecified with intoxication, uncomplicated
292.84	F19.94	Other psychoactive substance use, unspecified with psychoactive substance-induced mood disorder
292.85	F19.982	Other psychoactive substance use, unspecified with psychoactive substance-induced sleep disorder
292.89	F15.959	Other stimulant use, unspecified with stimulant-induced psychotic disorder, unspecified
292.89	F19.959	Other psychoactive substance use, unspecified with psychoactive substance-induced psychotic disorder, unspecified
292.9	F12.19	Cannabis abuse with unspecified cannabis-induced disorder
292.9	F19.99	Other psychoactive substance use, unspecified with unspecified psychoactive substance-induced disorder
304.1	F13.20	Sedative, hypnotic or anxiolytic dependence, unspecified
304.11	F13.20	Sedative, hypnotic or anxiolytic dependence, continuous
304.13	F13.21	Sedative, hypnotic or anxiolytic dependence, in remission
304.13	F13.21	Sedative, hypnotic or anxiolytic dependence, in remission
304.2	F14.20	Cocaine dependence, unspecified
304.2	F14.20	Cocaine dependence, uncomplicated
304.23	F14.21	Cocaine dependence, in remission
304.23	F14.21	Cocaine dependence, in remission
304.3	F12.20	Cannabis dependence, unspecified
304.31	F12.20	Cannabis dependence, continuous
304.33	F12.21	Cannabis dependence, in remission
304.4	F15.20	Amphetamine and other psychostimulant dependence, unspecified

© 2020 Palumbo SA et al. *JAMA Network Open.*

304.41	F15.20	Amphetamine and other psychostimulant dependence, continuous
304.43	F15.21	Amphetamine and other psychostimulant dependence, in remission
304.6	F19.20	Other specified drug dependence, unspecified
304.61	F19.20	Other specified drug dependence, continuous
304.63	F19.21	Other specified drug dependence, in remission
304.63	F19.21	Other psychoactive substance dependence, in remission
304.9	*	Unspecified drug dependence, unspecified
304.91	*	Unspecified drug dependence, continuous
304.92	*	Unspecified drug dependence, episodic
304.93	*	Unspecified drug dependence, in remission
305.2	F12.10	Cannabis abuse, unspecified
305.21	F12.10 or F12.90	Cannabis abuse, continuous
305.22	F12.10 or F12.90	Cannabis abuse, episodic
305.23	F12.11	Cannabis abuse, in remission
305.33	F16.11	Hallucinogen abuse, in remission
305.4	F13.10	Sedative, hypnotic or anxiolytic abuse, unspecified
305.41	F13.10	Sedative, hypnotic or anxiolytic abuse, continuous
305.42	F13.10	Sedative, hypnotic or anxiolytic abuse, episodic
305.6	F14.10	Cocaine abuse, unspecified
305.61	F14.10	Cocaine abuse, continuous
305.63	F14.11	Cocaine abuse, in remission
305.7	F15.10	Amphetamine or related acting sympathomimetic abuse, unspecified
305.7	F15.10	Other stimulant abuse, uncomplicated
305.73	F15.11	Amphetamine or related acting sympathomimetic abuse, in remission
305.8	F19.10	Antidepressant type abuse, unspecified
305.8	F19.10	Other psychoactive substance abuse, uncomplicated
305.8	F19.90	Other psychoactive substance use, unspecified, uncomplicated
305.9	F19.10 or F18.10	Other, mixed, or unspecified drug abuse, unspecified
305.91	F19.10 or F18.10	Other, mixed, or unspecified drug abuse, continuous
305.92	F19.10 or F18.10	Other, mixed, or unspecified drug abuse, episodic
305.93	F19.11 or F18.11	Other, mixed, or unspecified drug abuse, in remission
292.0 or 304.10	F13.239	Sedative, hypnotic or anxiolytic dependence with withdrawal, unspecified
292.0 or 304.60	F19.230	Other psychoactive substance dependence with withdrawal, uncomplicated

© 2020 Palumbo SA et al. *JAMA Network Open.*

304.1 or 304.11	F13.20	Sedative, hypnotic or anxiolytic dependence, uncomplicated
304.3 or 304.31	F12.20	Cannabis dependence, uncomplicated
304.4 or 304.41	F15.20	Other stimulant dependence, uncomplicated
304.6 or 304.61	F19.20	Other psychoactive substance dependence, uncomplicated
305.2 or 305.21 or 305.22	F12.10	Cannabis abuse, uncomplicated
305.21 or 305.2 or 305.22	F12.90	Cannabis use, unspecified, uncomplicated
305.4 or 305.41 or 305.42	F13.10	Sedative, hypnotic or anxiolytic abuse, uncomplicated
305.6 or 305.51 or 305.62	F14.10	Cocaine abuse, uncomplicated

© 2020 Palumbo SA et al. *JAMA Network Open.*

eTable 2. Demographic and Health Record Summary Data for GMMP-M and GMMP-V Subgroups

	GMMP-M N= 14,866	GMMP-V N=1,387	*P* Value		Test Statistic	Effect Size	95% CI
**Sample Demographics**							
**Male**	6,327	620					
**Female**	8,539	767	0.10		1.64	0.02	(0.0045, 0.0494)
**Age**	52.3	47.2	<0.001		15.60	5.15	(4.504, 5.799)
**BMI**	31.8	30.5	<0.001		5.75	1.27	(0.8374, 1.7040)
**Health Record Data**							
**EHR length in days**	4231	4004	<0.001		4.28	226	(122.8, 330.6)
**ER visits**	8.3	11.1	<0.001		-4.34	2.77	(1.518, 4.022)
**Daily MME**	52.0	48.8	0.01		2.44	3.20	(0.6336, 5.776)
**Psychiatric Codes**							
**Depression**	4902	591	<0.001		35.86	0.08	(0.0513, 0.1048)
**Anxiety**	5896	702	<0.001		42.09	0.09	(0.0606, 0.1150)
**Addiction Codes**							
**Alcohol**	424	74	<0.001		21.97	0.02	(0.0105, 0.0345)
**Nicotine**	4178	622	<0.001		139	0.15	(0.1211, 0.1747)
**Opioids**	243	51	<0.001		25.5	0.02	(0.0087, 0.0288)
**Other Substance Abuse**	451	130	<0.001		134	0.06	(0.0439, 0.0745)

***GMMP-M and GMMP-V groups were based on all available people with each designation and therefore do not represent matched groups.

© 2020 Palumbo SA et al. *JAMA Network Open.*

eTable 3. EHR Search Categories and Number of Patients With Each Category

	Patient Group		
	Controls	GMMP-M	GMMP-V
**Vocational Interference Due to Drug Use or Pain**	16	21	7
**Disabled**	34	58	44
**Weaning Described as Unsuccessful or Difficult**	1	10	13
**Positive Tox Screen for Opioids other than Prescribed**	6*	7*	30*
**Lost Pills**	3	23	31
**Multiple Opioid Prescribers**	11	11	47
**Multiple Pharmacies**	2	6	10
**Early Prescription Refills**	9	52	64
**Opioid Overdose**	4	7	6
**Substance Abuse**	18	28	51
**Hazardous Situation as Result of Opioid**	4	20	23
**Interpersonal or Legal Issues as Result of Opioid**	4	19	14
**Medical Issues as Result of Opioid**	15	41	29
**Craving**	0	2	4
**Provider Mentioned Drug-seeking Behavior**	5	10	21

*These numbers correspond to individuals that tested positive for an unprescribed opioid. Toxicology screens that are negative for a prescribed opioid and/or positive for unprescribed controlled substances are also considered violations. For complete breakdown of other toxicology screen violations, see eTable 4. Gray boxes indicate other categories that would violate the terms of the GMMP contract.

© 2020 Palumbo SA et al. *JAMA Network Open.*

eTable 4. Specific Toxicology Screen Violations by Subtype

	Controls	GMMP-M	GMMP-V
**Positive for unprescribed opioid**	6	7	30
**Positive for unprescribed non-opioid**	15	16	52
**Negative for prescribed opioid**	0	13	48
**Non-compliance with urine tox screen**	0	0	8
Positive for unprescribed medication*	17	20	64
Negative for prescribed medication*	0	17	51
Both (+) for unprescribed and (-) for prescribed	0	10	35

*Patients sometimes tested positive for more than one substance, thus values for all patients that test positive for unprescribed opioids/non-opioids, for example, do not add up to total # in 'positive for unprescribed medication'.

© 2020 Palumbo SA et al. *JAMA Network Open.*

eTable 5. Frequency of Positive Search Criteria

# of Positive Categories	Patient Group		
	Controls	GMMP-M	GMMP-V
**0**	124	11	0
**1**	50	18	13
**2**	11	19	17
**3**	6	18	22
**4**	4	12	13
**5**	4	2	13
**6**	1	5	6
**7**	0	9	6
**8**	0	3	7
**9**	0	1	1
**10**	0	1	1
**11**	0	1	1
**12**	0	0	0
**13**	0	0	0
**14**	0	0	0
**15**	0	0	0

© 2020 Palumbo SA et al. *JAMA Network Open.*
